# Liquid Crystal@Nanosilver Catalytic Amplification—Aptamer Trimode Biosensor for Trace Pb^2+^

**DOI:** 10.3390/ijms24032920

**Published:** 2023-02-02

**Authors:** Yiyi Shu, Sha Li, Chongning Li, Aihui Liang, Zhiliang Jiang

**Affiliations:** 1 School of Public Health, Guilin Medical University, Guilin 541199, China; 2 Guangxi Key Laboratory of Environmental Pollution Control Theory and Technology, Guangxi Normal University, Guilin 541006, China

**Keywords:** LC@AgNP catalytic amplification, AgNPs trimode indicator reaction, aptamer, inorganic pollutants

## Abstract

Liquid crystals (LCs) are a very important display material. However, the use of LC, especially LC-loaded nanoparticles, as a catalyst to amplify the analytical signal and coupled with specific aptamer (Apt) as a recognition element to construct a highly sensitive and selective three-mode molecular spectral assay is rarely reported. In this article, five LCs, such as cholesteryl benzoate (CB), were studied by molecular spectroscopy to indicate the liquid crystal nanoparticles in the system, and highly catalytic and stable CB loaded-nanosilver (CB@AgNPs) sol was prepared. The slope procedure was used to study the catalysis of the five LCs and CB@AgNPs on the new indicator reaction between AgNO_3_ and sodium formate (Fo) to produce silver nanoparticles (AgNPs) with a strong surface plasmon resonance absorption (Abs) peak at 450 nm, a resonance Rayleigh scattering (RRS) peak at 370 nm and a surface enhanced Raman scattering (SERS) peak at 1618 cm^−1^ in the presence of molecular probes. By coupling the new CB@AgNPs catalytic indicator reaction with the Apt reaction, a new CB@AgNPs catalytic amplification-SERS/RRS/Abs trimode biosensoring platform was constructed for detecting inorganic pollutants, such as Pb^2+^, Cd^2+^, Hg^2+^ and As^3+^.

## 1. Introduction

Liquid crystals (LC) are a kind of intermediate state polymer ordered material that combined the properties of crystal and liquid. They can have fluidity like a liquid, but they also have anisotropy similar to crystal [1]. Moreover, due to the elastic strain of the LC molecules, the small changes in the geomorphic surface could be further amplified to tens of microns [2]. After amplifying this small change and further converting it into a light signal observable under a polarizer, an LC sensor could be established. Compared with traditional analysis methods, it could easily detect analytes, such as enzyme activity [3,4] and trace elements [5], and has high sensitivity and does not require expensive equipment. Zhi [6] used liquid crystal (LC) trans, trans-4-(3,4-difluorophenyl)-4′-n-pentylbicyclohexyl (DP) to establish a simple and sensitive RRS energy transfer (RRS-ET) method for the determination of trace Cr^6+^ in water samples. Wang [7] realized the open-eye detection of kanamycin with LC film on a glass scaffold by a cross-polarizer based on the directional alignment changes of the LC surface caused by an Apt reaction. Wang [8] used a polarized light microscope to detect cocaine by using the conformational changes of the Apt on the water/LC interface. Verdian [9] demonstrated a novel LC-based sensor platform as a labeling free and rapid detection biosensor for PCB77, in which the limit was 1.5 × 10^−5^ μg/L. Nanomaterials, as new materials with at least 1–100 nm one-dimensional size, have been widely used in various fields. The combination of nanotechnology and LC for the determination of trace substances has been reported. The detection of amino acids, organophosphorus pesticides and enzymes by nanogold—signal amplification and nickel nanoparticles combined with an LC sensor is simple and sensitive. The nanoparticles loaded in liquid crystal (LC) media have emerged as an exciting field of research in recent years, because of these potentials for application and ease in controlling the orientation of the host and self-assembled structure by external stimulus [10]. Pourmostafa [11] found that 6CHBT doped with MgO nanoparticles will enhance the Kerr constant and birefringence effect. Mostafa et al. [12] studied the cholesteric liquid crystals (CLCs) that exhibit bistable properties. As far as we know, most of the research is at the physical level and there are no reports on the CB@AgNPs catalytic amplification and its application in the detection of trace inorganic pollutants by the aptamer SERS/RRS/Abs trimode biososensing platform.

SERS is a simple and highly sensitive molecular spectroscopy technique that has been applied in many frontier fields. We knew that some small organic compounds and metal ions have small scattering cross-sections, and their SERS method have low sensitivity and selectivity. It is of great significance to couple SERS with some selective biochemical reactions, such as Apt and enzyme reactions [13]. Liu [14] established a SERS detection method for determining 10 pm-1 μM insulin-like growth factor 2 receptor protein based on Apt reaction and gold/silver nano substrate. Liang [15] detected 1.3-16 pM Pb^2+^ by SERS and RRS using the strong catalysis of the prepared AuNPs and C_2_H_2_O_2_-HAuCl_4_ nanoreaction. Yu [16] used AuNFs with the SERS sol substrate and TMB_OX_ with the probe and detected iodide ions by the SERS method. Different than SERS, RRS is elastic scattering, with advantages of high sensitivity and simplicity. RRS has been used in the detection of pesticides, metal ions and other pollutants, especially when combined with nanoparticles as the probe. El Kurdi [17] reported that RRS signals of AuNPs could be amplified >10 times by growing into gold nanowires (AuNPs). Patra [18] successfully synthesized polyethylene glycol block–polypropylene glycol block–polyethylene glycol (F-108) functionalized AuNPs by using an RRS spectral probe to detect enzymes and labeled free sugars in serum samples. El-Kurdi [19] published the progress of chemical sensing and biosensing of gold and silver nanoparticles in RRS technology. Li [20] applied a multifunctional AuNPs probe based on the anti-EGFR Apt-and anti-EGFR antibody (Ab) to EGFR-positive cancer cells. When the probe was mixed with Eca109 ESCC cancer cells, a significant increase in RRS intensity was observed. The dynamic range of the probe for Eca109 cells was 1.0 × 10^2^–5.0 × 10^5^ cell·mL^−1^, and the detection limit was 20 cell·mL^−1^. Due to the advantages and disadvantages of various analysis methods, a single detection method might have problems, such as low sensitivity, poor stability and poor reproducibility. The dual mode and trimode methods could overcome these shortcomings and have attracted much attention. Based on the preparation of N-doped carbon points by microwave, Xi [21] combined with the Apt reaction of K^+^ and established a fast and efficient fluorescence-RRS dual mode coupling method for the determination of trace K^+^. Yao [22] prepared a new Au nanocluster-doped covalent organic framework (AuCOF) and established a dual-mode analysis platform of SERS/RRS based on an Apt-mediated COF nano-catalytic amplification signal strategy to detect ultra-trace small molecules. Huang [23] constructed an RRS/Abs dual-mode sensor using 4-heptylbenzoic acid (HPB), which could be used to determine 0.5ng/mL of oxytetracycline. Li [24] reported a new strategy of three-mode sensing based on nitrite colorimetric/fluorescence/SERS assembled by gold nanorods and azo–gold nanoparticles modulate based on the Griess reaction. Wang [25] developed a new three-mode Apt detection strategy for the detection of ultra-trace As^+^ based on the Apt_As_-As^+^ reaction mediating a CDAu-HAuCl_4_-fructose nanoreaction and the products of AuNPs as SERS/RRS/Abs trifunctional indicator nanoprobes. Compared to the reported nanocatalysts, such as metal, oxide, carbon dot, COF and MOF nanoparticles, the LC molecule has a stable and clear structure. Furthermore, the LC forms a nanoparticle catalyst by simple heating, which can be coupled with a catalytic indicator reaction. These advantages attract us to use it to amplify the trimode molecular signal to determine the inorganic pollutants in environmental water and agricultural product samples.

Inorganic pollutants, such as Pb(II), are important pollutants with multiple sources of pollution, and they exist in water, atmosphere or biota, and are extremely harmful to the human body. Pb(II) and its compounds enter the human body and have varying degrees of harmful effects on various cells and systems of the human body, damaging the human hematopoietic system, nervous system and kidneys [26,27], and causing various diseases. Therefore, it is of great significance to detect Pb^2+^ for environmental protection, agricultural product and human health. At present, there are several methods to detect Pb^2+^, such as atomic absorption spectrometry (AAS), inductively coupled plasma mass spectrometry (ICP-MS), fluorescence spectrometry (FL) and spectrophotometry [28,29,30]. Wang [31] adopted the colorimetric method to carry out semi-quantitative, visual and sensitive morphological analysis of Pb^2+^ in actual samples with double emission carbon points (CD), and the detection limit was 2.89 nM. Zhuang [32] combined DNA-based HCR with a specific DNAzyme to establish a new magnetic-controlled electrochemical DNA biosensor for the detection of 0.1–75 nM Pb^2+^ with a detection limit of 37 pM. However, these biosensor methods were relatively complex and time-consuming. AAS and ICP-MS require pretreatment and expensive equipment. FL, colorimetry and spectrophotometry are simple to operate, but have low sensitivity. Based on this, a new nanosilver sol SERS/RRS/Abs biosensoring platform was established for the determination of trace inorganic pollutants by combining CB@AgNPs catalytic amplification with Apt technology, which opened up a new field of LCs analysis and application.

## 2. Results and Discussion

### 2.1. Analysis Principle

**Figure 1:**
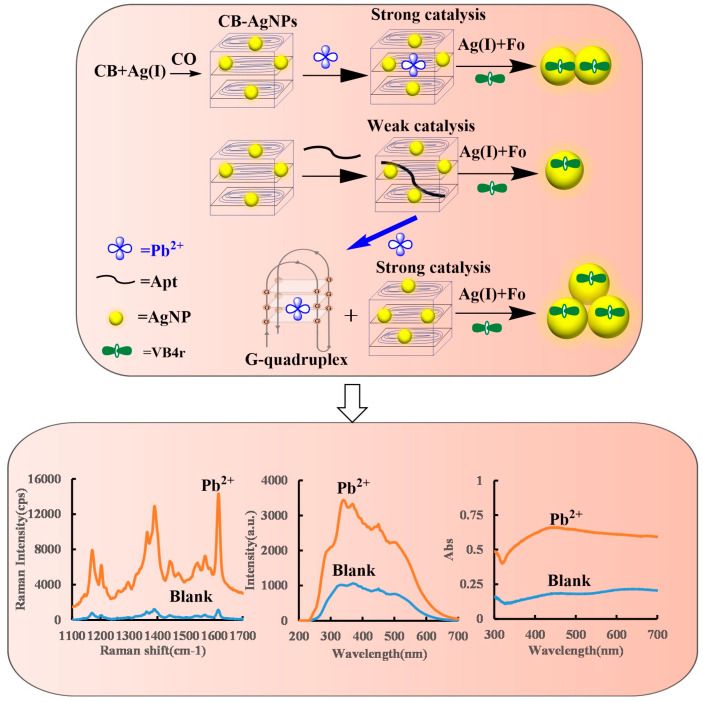
SERS/RRS/Abs trimode detection of inorganic pollutant Pb^2+^ coupled with CB@AgNPs catalysis amplification with the Apt.

The CB belonged to cholesterol LC, and its shape showed a spontaneous helical structure arrangement of layered molecules [33,34]. When AgNPs were loaded in the CB, it formed CB@AgNPs that could effectively catalyze the AgNO_3_-Fonanosilver indicator reaction, and the produced AgNPs caused the increase of the SERS/RRS/Abs signal. When the Apt was added before the reaction, the Apt could adsorb on the CB@AgNPs surface to inhibit its catalytic performance, so the AgNO_3_-Fo reaction was difficult to carry out, and the SERS/RRS/Abs signal of the system was low. When only the Pb^2+^ was added, the AgNO_3_-Fo reaction could not be enhanced. Formation of G-quadruplex structures requires the Apt sequence and the related cation. When the Pb^2+^ and the Apt were added, the Apt sequence of 5′–GGGTGGGTGGGTGGGT–3′ reacted with Pb^2+^ to form the G-quadruplex [35,36]. The Apt sequence of ATG CAA ACC CTT AAG AAA GTG GTC GTC CAA AAA ACC ATT G; the Apt sequence of GGA CTG TTG TGG TAT TAT TTT; and the Apt sequence of 5′–TTT CTT CTT TCT TCC CCC CTT GTT TGT TGT TT–3′ reacted with As^3+^, Cd^2+^ and Hg^2+^, respectively, to form the Apt complex but could not produce the G-quadruplex. The CB@AgNPs recovered their catalytic activity and generated more AgNPs-indicating components. The SERS/RRS/Abs signal was linearly enhanced, and a three-mode biosensoring platform for the determination of inorganic pollutants such as Pb^2+^ could be constructed (Figure 1). 

### 2.2. Nanomaterial Characterization

#### 2.2.1. Transmission Electron Microscope of AgNPs and CB@AgNPs

**Figure 2:**
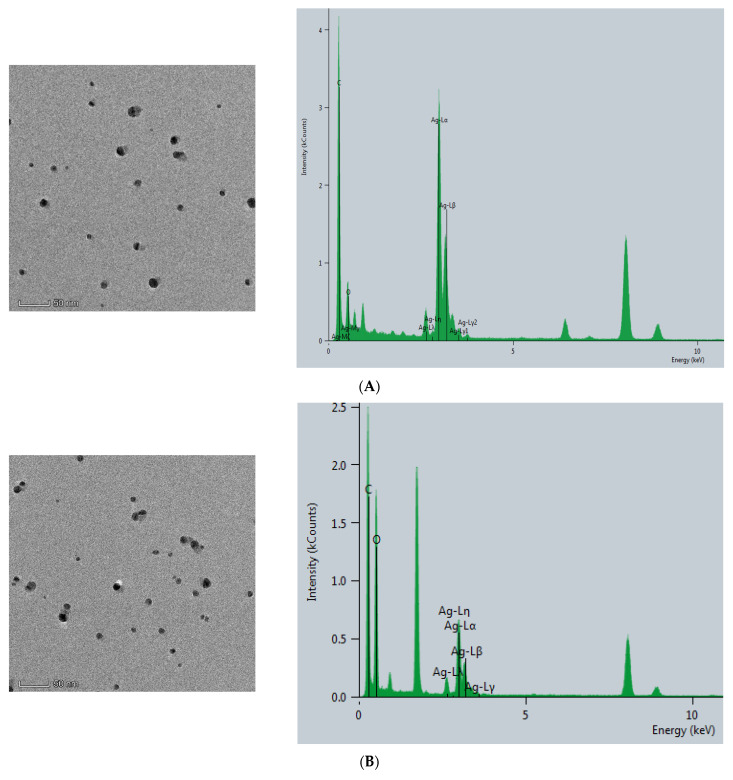

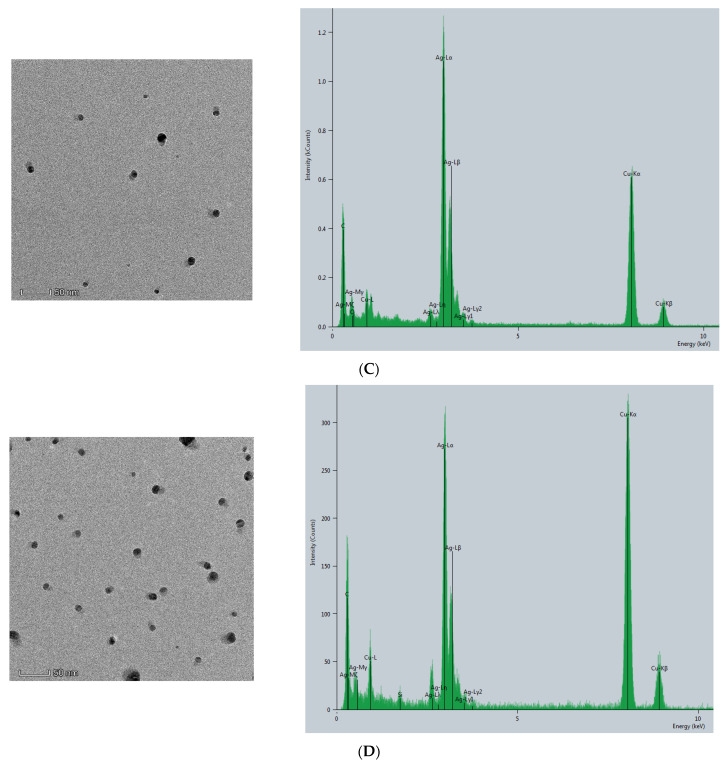
Transmission electron microscope of AgNPs and CB@AgNPs. (**A**): CB@AgNPs; (**B**): AgNPs; (**C**): 0.667 nmol/L Apt_Pb_ + 73.98 μmol/L NaAc-HAc + 1.33 µmol/L CB + 1.33 mmol/L AgNO_3_ + 0.1 mol/L Fo; (**D**): C + 0.133nmol/L Pb^2+^.

The CB@AgNPs and AgNPs were prepared according to the experimental method. Then, the Thermofisher Talos F200s transmission electron microscope (TEM) was used to record the image and energy spectra of the sample. As shown in (Figure 2A), AgNP has a spherical structure, and their average particle size was about 20 nm. Compared with AgNPs, CB@AgNPs are composed of evenly distributed C, O and Ag elements, which exhibit two peaks at 2.5 and 3 keV for Ag. It can be seen from its energy spectrum that the doping of AgNPs does not significantly change the structure of CB. It can also clearly be seen from the EDS mapping that AgNPs have been doped in CB (Figure 2B). In addition, the TEM analysis of AgNPs produced by the Apt_Pb_-CB@AgNPs-Fo-AgNO_3_ reaction were recorded (Figure 2C). When the Pb^2+^ was not added, fewer AgNPs were formed, and their average particle size was 20 nm. When Pb^2+^ was added, a large number of AgNPs particles were generated by the reaction, and their average particle size was about 40 nm (Figure 2D). 

#### 2.2.2. Molecular Spectrum, Zeta Potential and Particle Size Distribution of CB, AgNPs and CB@AgNPs

**Figure 3:**
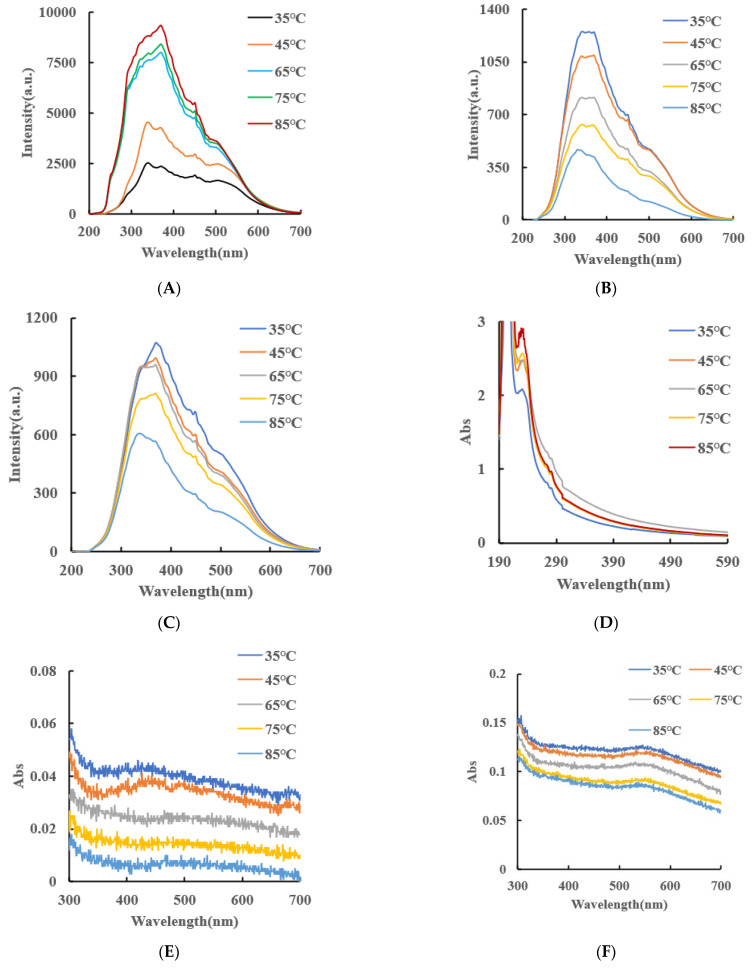

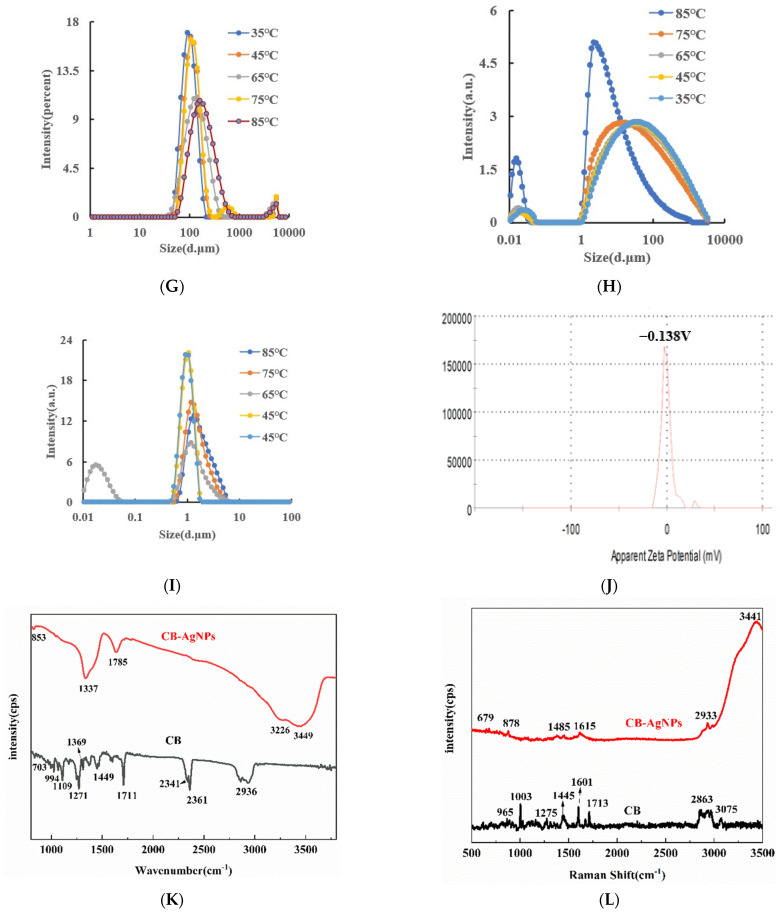
Molecular spectroscopy, particle size analysis and surface charge analysis of small molecule CB/CB@AgNPs/AgNPs. (**A**–**C**): Represents the RRS spectra of 0.01mmol/L CB/CB@AgNPs/AgNPs at different temperatures, respectively; (**D**–**F**): Abs spectra of 0.01mmol/L CB/CB@AgNPs/AgNPs at different temperatures, respectively; (**G**–**I**): Particle size distribution of 0.01mmol/L CB/CB@AgNPs/AgNPs at different temperatures, respectively; (**J**): 0.01mmol/L CB (zeta potential = −0.138mV); (**K**): Normal Raman spectrum (CB/CB@AgNPs); (**L**): FITR spectrum (CB/CB@AgNPs).

RRS spectroscopy is a simple and sensitive technique for studying the scattering properties of nanoparticles. Under the condition of Volt = 350 V and excitation slit = emission slit = 5 nm, the RRS spectra of CB, AgNPs and CB@AgNPs at different temperatures can be obtained easily and quickly. Figure 3A shows that when temperature was less than 45 °C, CB exhibits the strongest RRS peak at 340 nm and there are nanoparticles at room temperature. When the temperature was higher than 65 °C, the strongest RRS peak changed to 370 nm. Both peak RRS values all enhanced greatly with the increasing temperature, indicating that the number of formed CB nanoparticle increased. In addition, the red-shift of the strongest RRS peak was due to the increasing size. Figure 3B and C show that the RRS intensity of CB@AgNPs and AgNPs decreased with increasing temperature. Figure 3D is the UV absorption spectrum of CB. The Abs peak at 226 nm enhanced with temperature increasing that was related to the changes of the CBN number and size. Figure 3E,F is the UV absorption spectrum of CB@AgNPs and AgNPs, respectively. The Abs signal value peak at 446 nm decreased with increasing temperature.

Laser scattering is a good and reliable technique to study the particle size distribution in a solution because the conditions of the system were not destroyed. The particle size distribution of the CB materials at different temperatures was studied by a laser scattering nanoparticle analyzer. It can clearly be observed from Figure 3G that, in a certain temperature range (35–85 °C), the diameter of the CB increases from 101, 116, 123 and 154 to 171 nm when the temperature increased. This was because the CB molecules were gradually aggregated into the nanoparticles with a larger size as the temperature increased. Figure 3J shows that the surface of the CBN material is negatively charged, and the Zeta potential is −0.138 mV, indicating it was of good stability. The particle size distribution of the CB@AgNPs and AgNPs materials at different temperatures was studied by laser scattering nanoparticle analyzer. It can be clearly observed from Figure 3H,I that, in a certain temperature range (35–85 °C), the diameter of CB@AgNPs and AgNPs decreases when the temperature increases.

Using nanosilver as a SERS base, we studied the Raman spectrum of the CB solution. The results show that there is no obvious characteristic peak. After that, 0.1 m of a NaCl solution was added as sensitizer, but there was still no obvious characteristic peak on the spectrum. Therefore, a small amount of solid CB powder was dried by rectangular glass plate, then the other glass sheet was rolled, the CB powder was dispersed, and then placed in a Raman measurement instrument to obtain the CB normal Raman spectra. The normal Raman spectra of the CB solid (Figure 3K) show 8 peaks, of which 865 cm^−1^ is ring “respiration”; 1003 cm^−1^ is the triangular ring “breathing”; 1275 cm^−1^ is the ring vibration of a para-disubstituted benzene ring; 1445 cm^−1^ is a ring stretch; 1601 cm^−1^ is caused by the expanded ring peak (double peak) of benzene derivatives; 854, 1154 and 1284 cm^−1^ are attributed to the ring vibration of the para-disubstituted benzene ring; 1608 cm^−1^ is caused by the expanded ring peak (double peak) of the benzene derivatives; 1713 cm^−1^ is an extension of the C-O bond; and 2864 cm^−1^ and 3075 cm^−1^ were attributed to CH_3_ stretching and aromatic C-H stretching, respectively. Using nanosilver as an SERS base and 0.1 m of a NaCl solution added as a sensitizer, we studied the Raman spectrum of the CB@AgNPs solution. The results (Figure 3K) show that there are 7 peaks, of which 436 cm^−1^ and 679 cm^−1^ are skeletal bending; 878 cm^−1^ is ring “respiration”; 1485 cm^−1^ is a ring stretch; 1615 cm^−1^ is caused by the expanded ring peak (double peak) of the benzene derivatives; 2933 cm^−1^ is attributed to aromatic C-H stretching; and 3441 cm^−1^ is attributed to aromatic O-H stretching. 

Figure 3L shows the infrared spectrum of CB, in which the characteristic peaks at 994 cm^−1^ are caused by the out-of-plane curvature of C-H, and 1109 cm^−1^ is the C-O bond caused by expansion and contraction; 1271 cm^−1^ is an in-plane flexion caused by a para-substituted phenyl group; 1369, 1449 and 1711 cm^−1^ are extensions of C-O bonds; 2341 and 2361 cm^−1^ are vibration peaks caused by triple bonds and cumulative double bonds; and 2936 cm^−1^ is the stretching vibration peak caused by CH. Figure 3L shows the infrared spectrum of CB@AgNPs, in which the characteristic peaks at 853 cm^−1^ are caused by the out-of-plane curvature of C-H; 1337 is extensions of the C-O bonds; and 3226 and 3449 cm^−1^ are the stretching vibration peak caused by CH.

To test the stability of AgNPs and CB@AgNPs, their stability in NaCl and over time were investigated, respectively. A total of 200 μL of 1 mmol/L AgNPs and CB@AgNPs were placed in a 10 mL plug calibrated tube. Different volumes of a 1 mol/L NaCl solution were added, and the volume was fixed to 1.5 mL. After the mixture was evenly mixed, the RRS signal was measured (Figure S1A,B). Figure S1A shows that with the increase of days, the RRS signal values of AgNPs and CB@AgNPs were in a stable state. In addition, the stability of these catalytic materials was investigated in different concentrations of NaCl solutions, it was also found that the AgNPs materials were unstable in NaCl solutions with different concentrations. The signal value would rise first and then increased slightly, which might be caused by the AgNPs aggregation in salt solutions. The signal values of CB@AgNPs in the low salt solution were very stable. With the increase of the NaCl concentration, the signal values increased slightly, which might be caused by the AgNPs aggregation in the solution. The results show that CB@AgNPs was relatively stable in salt solution for a long time, while AgNPs had poor stability and tended to aggregate in a salt solution. When the AgNPs was mixed with CB, it could be uniformly loaded in the CB, which had a greater improvement in the stability of the salt than AgNPs.

### 2.3. SERS Spectra of a Nanocatalytic System

**Figure 4:**
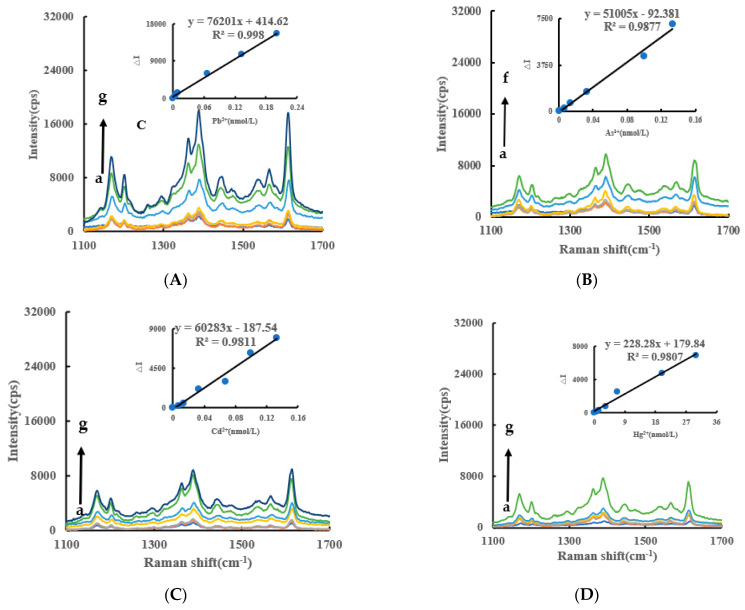
SERS spectra of the CB@AgNPs -Fo-AgNO_3_-Apt-inorganic pollutants system. (**A**): a–g: (0, 4.47 × 10^−3^, 6.7 × 10^−3^, 8.94 × 10^−3^, 6.7 × 10^−2^, 0.133, 0.201) nmol/L Pb^2+^ + 0.667 nmol/L Apt_Pb_ + 73.98 µmol/L NaAc-Hac + 1.33 µmol/L CB@AgNPs + 1.33 mmol/L AgNO_3_ + 0.1 mol/L Fo + 0.67 µmol/L VB4r + 0.067 mol/L NaCl; (**B**): a–f: (0, 6.7 × 10^−3^, 1.33 × 10^−2^, 3.3 × 10^−2^, 0.1, 0.133) nmol/L As^3+^ + 0.667 nmol/L Apt_As_ + 73.98 µmol/L NaAc-Hac + 1.33 µmol/L CB@AgNPs + 1.33 mmol/L AgNO_3_ + 0.1 mol/L Fo + 0.67 µmol/L VB4r + 0.067 mol/L NaCl; (**C**): a–g: (0, 6.7 × 10^−3^, 1.33 × 10^−2^, 6.7 × 10^−2^, 3.3 × 10^−2^, 0.1, 0.133) nmol/L Cd^2+^ + 0.667 nmol/L Apt_Cd_ + 73.98 µmol/L NaAc-Hac + 1.33 µmol/L CB@AgNPs + 1.33 mmol/L AgNO_3_ + 0.1 mol/L Fo + 0.67 µmol/L VB4r + 0.067 mol/L NaCl; (**D**): a–g: (0, 0.67, 1.33, 3.3, 6.7, 20,30) nmol/L Hg ^2+^ + 0.667 nmol/L Apt_Hg_ + 73.98 µmol/L NaAc-Hac + 1.33 µmol/L CB@AgNPs + 1.33 mmol/L AgNO_3_ + 0.1 mol/L Fo + 0.67 µmol/L VB4r + 0.067 mol/L NaCl.

Under experimental conditions, AgNO_3_ was reduced to AgNPs by Fo slowly, and fewer AgNPs were generated in the system. When the CB@AgNPs/AgNPs/LC catalysts were added to the system, the reaction of the AgNPs were significantly accelerated, and the generated AgNPs were not only the indicator component, but also the sol substrate. When VB4r was added as a probe, a strong SERS peak appeared at 1618 cm^−1^. When Apt_Pb_ was present, it was adsorbed to the surface of the CB@AgNPs catalyst, the catalytic activity decreased, and the generated AgNPs decreased, which led to the decrease of the SERS signal of the system. When Pb^2+^ was added, the Apt_Pb_ combined with the Pb^2+^ to generate a stable G-tetrad structure, the catalytic activity of the CB@AgNPs was restored, the generated AgNPs increased, and the SERS signal was linearly enhanced. Within a certain concentration range, the SERS signal of the system was gradually enhanced with the increase of the concentration of the substance to be measured, and the SERS peak of the system at 1615 cm^−1^ increased linearly (Figure 4A). The other inorganic pollutants to be measured, namely As^3+^, Cd^2+^ and Hg^2+^, were less sensitive than Pb^2+^ (Figure 4B–D). From the slope of the linear curve of the CB@AgNPs/AgNPs/LC concentration and the SERS peak, we know that the slope of the CB@AgNPs system was the largest and its catalytic effect was the strongest (Figure 4A, Figure S2A–E).

### 2.4. RRS Spectra of a Catalytic Amplification System

**Figure 5:**
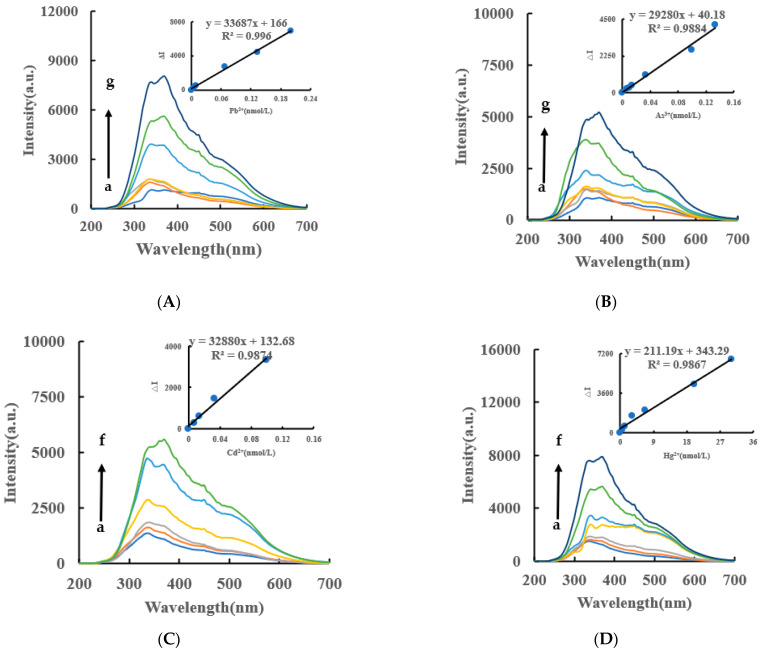
RRS spectra of CB@AgNPs -Fo-AgNO_3_-Apt- inorganic pollutants system RRS spectrum system. (**A**): a–g: (0, 4.47 × 10^−3^, 6.7 × 10^−3^, 8.94 × 10^−3^, 6.7 × 10^−2^, 0.133, 0.201) nmol/L Pb^2+^ + 0.667 nmol/L Apt_Pb_ + 73.98 µmol/L NaAc-Hac + 1.33 µmol/L CB@AgNPs + 1 mmol/L AgNO_3_ + 0.1 mol/L Fo; (**B**): a–g: (0, 6.7 × 10^−3^, 1 × 10^−2^, 1.33 × 10^−2^, 3.3 × 10^−2^, 0.1, 0.133) nmol/L As^3+^ + 0.667 nmol/L Apt_As_ + 0.667 nmol/L Apt_Pb_ + 73.98 µmol/L NaAc-Hac + 1.33 µmol/L CB@AgNPs + 1 mmol/L AgNO_3_ + 0.1 mol/L Fo; (**C**): a–f: (0, 6.7 × 10^−3^, 1.33 × 10^−2^, 3.3 × 10^−2^, 0.1, 0.133) nmol/L Cd^2+^ + 0.667 nmol/L Apt_Cd_ + 0.667 nmol/L Apt_Pb_+73.98 µmol/L NaAc-HAc+1.33 µmol/L CB@AgNPs + 1 mmol/L AgNO_3_ + 0.1 mol/L Fo; (**D**): a–f: (0, 0.67, 1.33, 3.3, 6.7, 20, 30) nmol/L Hg ^2+^ + 0.667 nmol/L Apt_Hg_ + 0.667 nmol/L Apt_Pb_ + 73.98 µmol/L NaAc-Hac + 1.33 µmol/L CB@AgNPs + 1 mmol/L AgNO_3_ + 0.1 mol/L Fo.

VB4r was not added to the RRS and it is more simple than the SERS. Under experimental conditions, the AgNPs generated by the Apt_Pb_-CB@AgNPs-Fo-AgNO_3_-Pb^2+^ system had a strong RRS peak at 370 nm. Within a certain concentration range, the RRS signal of the system was gradually enhanced with the increase of the Pb^2+^ concentration (Figure 5A), based on which a new RRS method for detecting Pb^2+^ could be established. The RRS spectra of Apt-CB@AgNPs-Fo-AgNO_3_-As^3+^/Cd^2+^/Hg^2+^ are shown in Figure 5B–D. The Apt_Pb_- AgNPs/LC -Fo-AgNO_3_-Pb^2+^ system also has an RRS peak (Figure S3A–F).

### 2.5. Abs Spectra of a Catalytic Amplification System

**Figure 6:**
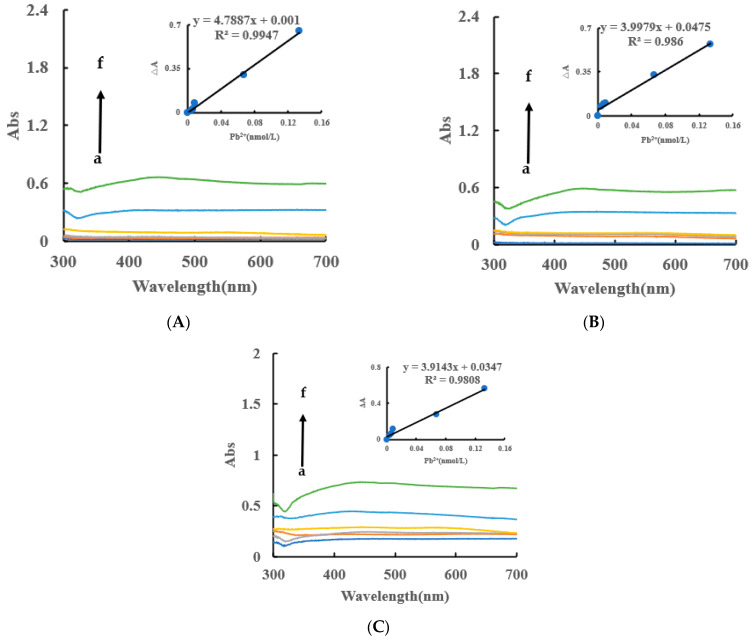
Abs spectra of an Apt_Pb_-CB@AgNPs -Fo-AgNO_3_-Pb^2+^ system. (**A**): a–f: (0, 4.47 × 10^−3^, 6.7 × 10^−3^, 8.94 × 10^−3^, 6.7 × 10^−2^, 0.133) nmol/L Pb^2+^ + 0.667 nmol/L Apt_Pb_ + 73.98 µmol/L NaAc-Hac + 1.33 µmol/L CB@AgNPs + 1 mmol/L AgNO_3_ + 0.1 mol/L Fo; (**B**): a–f: (0, 4.47 × 10^−3^, 6.7 × 10^−3^, 8.94 × 10^−3^, 6.7 × 10^−2^, 0.133) nmol/L Pb^2+^ + 0.667 nmol/L Apt_Pb_ + 0.667 nmol/L Apt_Pb_ + 73.98 µmol/L NaAc-Hac + 1.33 µmol/L AgNPs + 1 mmol/L AgNO_3_ + 0.1 mol/L Fo; (**C**): a–f: (0, 4.47 × 10^−3^, 6.7 × 10^−3^, 8.94 × 10^−3^, 6.7 × 10^−2^, 0.133) nmol/L Pb^2+^ + 0.667 nmol/L Apt_Pb_ + 0.667 nmol/L Apt_Pb_ + 73.98 µmol/L NaAc-Hac + 1.33 µmol/L CB + 1 mmol/L AgNO_3_ + 0.1 mol/L Fo.

Under the experimental conditions, the AgNPs produced by the Apt_Pb_-CB@AgNPs-Fo-AgNO_3_-Pb^2+^ system had an absorption peak at 450 nm, which was corresponded to the surface plasmon resonance absorption peak of AgNPs. Within a certain concentration range, as the concentration of Pb^2+^ increased, the absorbance of the system gradually increased, and an absorption peak appeared at 450 nm (Figure 6A). Similarly, the AgNPs/CB system has an absorption peak at 430 nm, and the AgNPs, OA and HA systems have an absorption peak at 450 nm (Figure 6B,C). In addition, the DB and DE nanocatalytic analytical systems have an absorption peak at 440 nm (Figure S4A–F). From this data, we can determine that the CB@AgNPs catalytic analytical system was the most sensitive.

### 2.6. CB@AgNPs and LC Catalytic Mechanism

Under the catalysis of CB@AgNPs, AgNO_3_ was reduced by Fo to produce AgNPs. Within a certain concentration range, as the concentration of the catalyst increased, the catalytic ability increased, and the amount of the AgNPs generated by catalysis increased, which had a strong RRS peak at 370 nm. Table S1 shows that the slope of the working curve of the CB@AgNPs RRS system was seven times that of CB and two times that of AgNPs, respectively. It can be seen that the slope of the CB@AgNPs system is the largest, and most AgNPs are generated in the system. When Apt_Pb_ was added to the system, it could be adsorbed on the surface of the catalyst due to static electricity and molecular forces and inhibited its catalytic activity. With the increase of the Apt_Pb_ concentration, the concentration of the free catalyst in the system decreased, and the catalytic activity weakened, thus the AgNPs generated in the system decreased, and the RRS signal of the system weakened (Table S1). 

**Figure 7:**
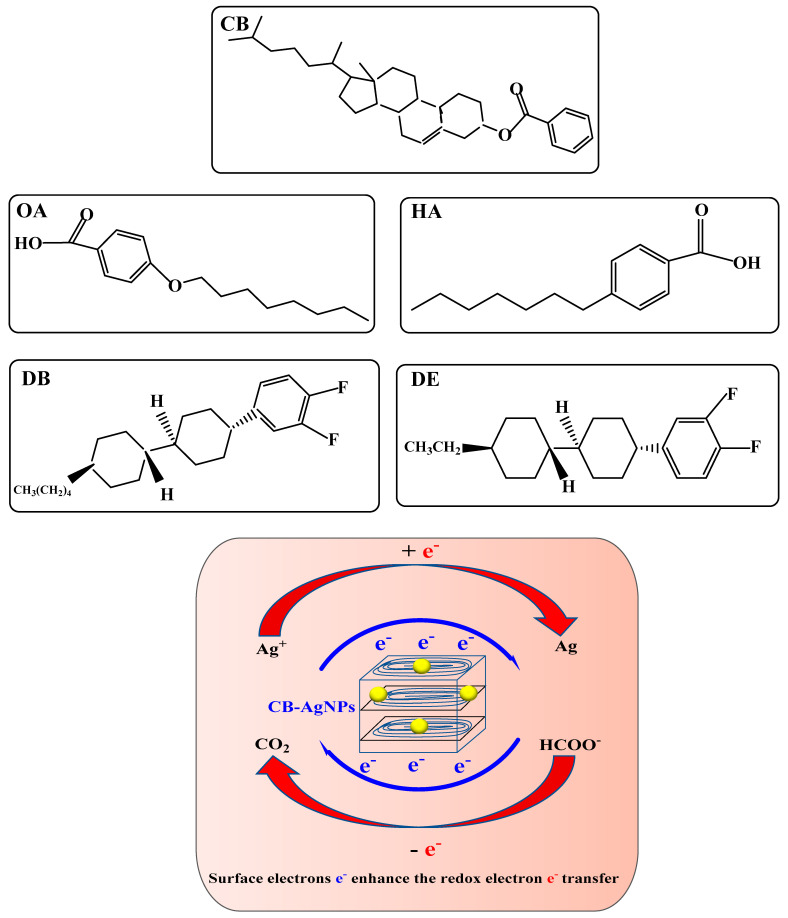
Catalytic enhancement mechanism of LCs and CB@AgNPs on the AgNO_3_-Fo nanoreaction.

Because the hydrogen bond association force in the cholesterol molecule is so strong when the solid reaches its melting point, the solid directly became an isotropic liquid, which means that cholesterol did not have an LC phase, and CB did have an LC phase. The ring system in the molecule is another important factor that determines whether the molecule has an LC phase. Molecules with aromatic rings do not necessarily have an LC phase, but molecules with an LC phase almost all have more than one aromatic ring. Like the five LC molecules and CB@AgNPs used in this experiment, when the solid melts into a liquid, the aromatic ring maintains the intermolecular relationship. The short-range attractive force prevents the molecule from turning into an isotropic liquid immediately. Common ring systems include saturated six rings and unsaturated benzene rings, or a combination of the two. The electrons in the saturated six-membered ring were σ electrons, which are not conjugated to each other, while the electrons in the benzene ring are π electrons, which have a conjugation effect. The conjugation of electrons has an important influence on the properties of LCs, especially the π electrons in the benzene ring, which help to promote electron transfer in redox reactions. The study finds that these five LCs and CB@AgNPs materials can catalyze the reduction of AgNO_3_ by Fo to generate AgNPs. Among the five types of LC and CB@AgNPs, the slope of the linear relationship between the CB@AgNPs concentration and the SERS/RRS/Abs is the largest, and it has the strongest catalytic effect on the AgNO_3_-Fo system. This is very much related to the molecular structure of CB (Figure 7) with the most π electrons and without an electron absorbing base F, and the higher LC phase temperature of 145–150 °C that makes it easy to form CB nanoparticles. Nanoparticles, such as metal nanozymes, semiconductor nanozymes, carbon dot nanozymes, metal organic framework nanozymes and covalent organic framework nanozymes, are rich in surface electrons, which are the driving force for promoting electron transfer in some redox reactions. The formate reduction of AgNO_3_ can slowly generate AgNPs and CO_2_. These five small molecules of LC and CB@AgNPs can be arranged and assembled into rod-shaped nanoparticles in an aqueous solution at 95 °C, and their surface is rich in surface electrons. Ag^+^ and HCOO^−^ can be adsorbed on the surface of LC and CB@AgNPs nanoparticles. This nano surface electron can accelerate the redox electron transfer between formic acid and silver ions, accelerate the formation of AgNPs and show a strong catalytic effect (Figure 7).

### 2.7. Optimization of Analysis Conditions

According to the experimental method, the SERS analysis system conditions were examined. When the experimental conditions were 0.667 nmol/L Apt_Pb_ (Figure S5A), 73.98 µmol/L NaAc-HAc (Figure S5B), 1.33 mmol/L AgNO_3_ (Figure S5C), 0.1 mol/L Fo (Figure S5D), a reaction temperature of 95 °C (Figure S5E), a reaction time of 20 min (Figure S5F), 1.33 µmol/L CB@AgNPs (Figure S5G), a standing time of 15 min (Figure S5H), 0.67 µmol/L VB4r (Figure S5I) and 0.067 mol/L NaCl solution (Figure S5J), the SERS signal of the analysis system was the largest; therefore, these conditions were selected as the SERS experimental conditions.

According to the experimental method, the RRS analysis system conditions were considered. When the experimental conditions were 0.667 nmol/L Apt_Pb_ (Figure S6A), 73.98 µmol/L NaAc-HAc (Figure S6B), 1 mmol/L AgNO_3_ (Figure S6C), 0.1 mol/L Fo (Figure S6D), a reaction temperature of 85 °C (Figure S6E), a reaction time of 15 min (Figure S6F), 1.33 µmol/L CB@AgNPs (Figure S6G) and a standing time of 15 min (Figure S6H), the ΔI of the analysis system was the largest; therefore, these conditions were selected for use. The conditions of the Abs method were the same as those of the RRS method.

### 2.8. Working Curve

Under the optimal experimental conditions, the different concentrations of Pb^2+^ were plotted to their corresponding ΔI/ΔA to obtain the working curve. It can be seen from Tables S2 and S3 that the slope of the working curve of the Apt-CB@AgNPs system was five times that of CB and 1.5 times that of AgNPs, respectively, indicating that it was the most sensitive. The linear equation was ΔI_1618cm−1_ = 76201C + 414.6, and the linear range was 4.47 × 10^−3^−0.201 nmol/L. Although the RRS/Abs had a short reaction time and low cost, its sensitivity was not as good as SERS (Table S2). In addition, other inorganic pollutants, such as As^3+^, Cd^2+^ and the Hg^2+^, can be also determined by a CB@AgNPs nanocatalytic-SERS/RRS assay platform (Table S3). Comparing with the reported methods for measuring lead ions [37,38,39,40,41,42] (Table S4), this method is a more sensitive molecular spectral method.

### 2.9. Influence of Interfering Ions

According to the experimental method, the interference of coexisting ions on the Apt_Pb_-CB@AgNPs-Fo-AgNO_3_-Pb^2+^ SERS determination of 0.1 nmol/L Pb^2+^ was investigated. The experimental results showed that the relative error is within ±10%, 1000 times Mg^2+^, Ba^2+^, Mn^2+^, Cr^6+^, Fe^2+^, Cr^3+^, Ca^2+^, BSA; 500 times Zn^2+^, NH^4+^, Co^2+^, serum protein, ascorbic acid, Al^3+^; and 100 times Fe^3+^, Hg^2+^, Cu^2+^, NO^2−^, has, PO_4_^3−^. This does not interfere with the measurement of Pb^2+^ (see Table S5). This method has good selectivity.

### 2.10. Sample Determination

Tap water and mineral water were measured at 100 mL each, and water samples were taken from pond water of the Yanshan Campus of Guangxi Normal University and wastewater of the Bokang Building Laboratory of Guangxi Normal University. The sample pretreatment: orange peels and preserved eggs were all purchased from local supermarkets. Different orange peels and preserved eggs were respectively weighed and cut into small pieces and dried 10 h in an oven at 105 °C. The dried sample was ground with an agate mortar. A total of 2.0 g (accurate to 0.1 mg) of each sample was weighed and put in a 25 mL conical flask with 10 mL of a mixed acid of HNO_3_ and HClO_4_ (V_1_:V_2_ = 4:1) and left overnight for about 12 h. The flask was placed on a power adjustable heating plate until white smoke appeared. When the solution became colorless and transparent or slightly yellow, it was removed from the heating plate and cooled. Then the volume was adjusted to 10 mL [43]. Finally, 100 μL of the samples were collected after filtration with a 0.45 μm microporous membrane. According to the experimental method, the Pb^2+^ was measured, and the known concentration of Pb(II) was added. The detection results are shown in Table S6. The content of Pb^2+^ in water samples was 0.033–0.3310 nmol/L, and the content of Pb^2+^ in food was 4.986–16.653 ng/g. The relative standard deviation (RSD) was 1.3–7.1%, and the recovery rate was 92.53–109.4%, showing good recovery and reproducibility.

## 3. Materials and Methods

### 3.1. Main Instruments

A Hitachi F-7000 fluorescence spectrophotometer (Hitachi High-Tech Company, Tokyo Japan); TU-1901 type dual-beam UV-Vis spectrophotometer (Beijing Puxi General Instrument Co., Ltd., Beijing, China); DXR Raman spectrometer (Thermo Fisher Scientific Company, Waltham, MA, USA); FEI Talos 200S radio microscope (Thermo Fisher Scientific company); 16K desktop centrifuge (Zhuhai Dark Horse Medical Instrument Co., Ltd., Zhuhai, China); Ultrasonic cleaner SK3300B (Shanghai Kedao Ultrasonic Instrument Co., Ltd., Shanghai, China); HH-1 electric heating constant temperature water bath (Shanghai Weicheng Instrument Co., Ltd., Shanghai, China); SYZ-550 type quartz sub-boiling water distiller (Jiangsu Jingbo Instrument Factory, Haian, China); DHG-9023A electric heating constant temperature blast drying oven (Shanghai Jinghong Experimental Equipment Co., Ltd., Shanghai, China); FB224 automatic internal school electronic analytical balance (Shanghai Sunny Hengping Scientific Instrument Co., Ltd., Shanghai, China); DF-101S-type heat-collecting constant temperature heating magnetic stirrer (Gongyi Yuhua Instrument Co., Ltd., Zhengzhou, China); Nanoparticle size and Zeta potential analyzer (Malvern, UK); Thermofisher Talos F200s transmission electron microscope (Thermo Fisher Scientific, USA); and S-4800 field emission scanning electron microscope (Hitachi High-Technologies Corporation, Japan/Oxford Corporation, Oxfordshire, UK) were used.

### 3.2. Main Reagents

Cholesteryl benzoate (CB), 4-octyloxybenzoic acid (OA), 4-heptylbenzoic acid (HA), trans, trans-4-(3,4-Difluorophenyl)-4′-n-pentylbicyclohexyl (DB) and trans-4-(3,4-difluorophenyl)-trans-4′-ethylbicyclohexane (DE) were obtained from Macklin Co., with an LC phase temperature of 145–150 °C,101–105 °C, 98 °C, 44–49 °C and 51–54 °C, respectively. A total of 0.049 g CB was dissolved in 10 mL of ethanol/water (1:1) to obtain a 0.01 mol/L CB solution. The 4-Hexyloxybenzoicacid(HB) without catalysis was obtained from Macklin. Apt_pb_: The Apt of the Pb(II) was synthesized by Biological Engineering Shenggong with the sequences of 5′–GGGTGGGTGGGTGGGT–3′; Apt_As_: The Apt of the As(III) was synthesized by Shanghai Sangon Biotech with a sequence of ATG CAA ACC CTT AAG AAA GTG GTC GTC CAA AAA ACC ATT G; Apt_Cd_: The Apt of Cd(II) was synthesized by Biological Engineering Shenggong with a sequence of GGA CTG TTG TGG TAT TAT TTT; Apt_Hg_: The Apt of Hg(II) was synthesized by Shanghai Sangon Biotech with a sequence of 5′–TTT CTT CTT TCT TCC CCC CTT GTT TGT TGT TT–3′; C_2_H_5_OH (AR, Guangdong Guanghua Technology Co., Ltd.); Pb(II) nitrate (AR, Shantou Xilong Chemical Factory Co., Ltd., Shantou, China); Victoria Blue 4r (VB4r); AgNO_3_ (AR, Guangdong Guanghua Technology Co., Ltd., Shantou, China); and sodium formate (Fo) were used. The reagents used were of analytical grade, and the experimental water was sub-boiling water.

Preparation of CB@AgNP: In a clean 100 mL conical flask, 5 mL of 0.01 mol/L AgNO_3_ was placed and added to 25 mL of water, then 5 mL of 0.01 mol/L CB and 20 mL of a saturated carbon monoxide solution were added, respectively, under the condition of magnetic stirring for 2 h to obtain1.0 mmol/L CB@AgNP nanosol.

Preparation of AgNPs: A total of 5 mL 0.01 mol/L AgNO_3_ was placed into a clean 100 mL conical flask, then 20 mL of a saturated carbon monoxide solution was added into the mixture at a constant speed in the stirred state and volumed to 50 mL, finally stirred for 1h to obtain 1mmol/L AgNPs.

### 3.3. Experimental Procedure

A 7.4 mM (pH 4.4) NaAc-HAc (88.8 mM NaAc) buffer solution, 200 µL of 0.1 mmol/L LC solution, 100 µL of 100 nM Apt_Pb_ and a certain concentration of the Pb(II) solution were added into a 5mL glass tube. After standing for 15 min, 200 µL 0.01 M AgNO_3_ and 150 µL 1 M Fo were added and shaken well, the volume was fixed to 1.5 ml, and heated in a 95 °C water bath for 20 min. After cooling with ice water, 100 µL of 1 mol/L NaCl solution and 100 µL of 10 µmol/L VB4r were added to the reaction solution. The Raman spectra were obtained by scanning under the conditions of the Raman spectrometer with a light source power of 2.0 mW and a slit of 25.0 nm. The SERS intensity at 1618 cm^−1^ (I_1618 cm−1_) was measured. Without Pb^2+^, the blank SERS signal (I_1618 cm−1_)_0_ was measured, then ΔI = I_1618cm−1_−(I_1618 cm−1_)_0_ was calculated. For the RRS and Abs analysis, no VB4r was added. 

## 4. Conclusions

The CB@AgNPs, AgNPs and LCs were characterized by a Zeta nanoparticle analyzer and molecular spectroscopy. The results show that the catalytic activity of LCs was evaluated by the slope procedure using molecular spectroscopy. It was found that CB@AgNPs has the strongest catalytic effect on the nanoreaction of AgNO_3_-Fo due to its rich surface electrons on the CB@AgNPs nanoparticles. This new and highly sensitive catalytic amplification indicator reaction was combined with a highly selective Apt reaction, with SERS/RRS/Abs molecular spectral technology, and a new three-mode biosensoring assay platform for the determination of Pb^2+^ was established. The SERS linear response range was 4.47 × 10^−3^ to 0.201 nmol/L and the detection limit was as low as 3 × 10^−3^ nmol/L. The Abs mode was low-cost and simple, without VB4r. This article has demonstrated that the SERS/RRS/Abs trimode method has high sensitivity and broad linear range for the SERS/RRS method, and the Abs/RRS method is of low-cost and simplicity. In addition, a nanocatalytic mechanism was proposed, and it was shown that the nano surface electrons enhanced the redox electron transfer of Ag^+^-Fo reaction. The method has also been successfully applied to determine trace Pb(II) in food and water samples. Similarly, the apt sensor can also be applied to other inorganic pollutants detection in environmental samples, which has great potential in biochemical analysis.

## Data Availability

No new data was created or analyzed in this study.

## Supplementary Materials

The following supporting information can be downloaded at: https://www.mdpi.com/article/10.3390/ijms24032920/s1.
