# Supplementary material for: Liquid Crystal@Nanosilver Catalytic Amplification—Aptamer Trimode Biosensor for Trace Pb2+
Source: Int J Mol Sci. 2023 Feb 2;24(3):2920. doi: 10.3390/ijms24032920 (PMC9917628; doi:10.3390/ijms24032920)
Supplement: Supplementary file 1 [file ijms-24-02920-s001.zip › ijms-2180705-supplementary.pdf]

International Journal of
Molecular Sciences

MDPI

Supplementary Materials

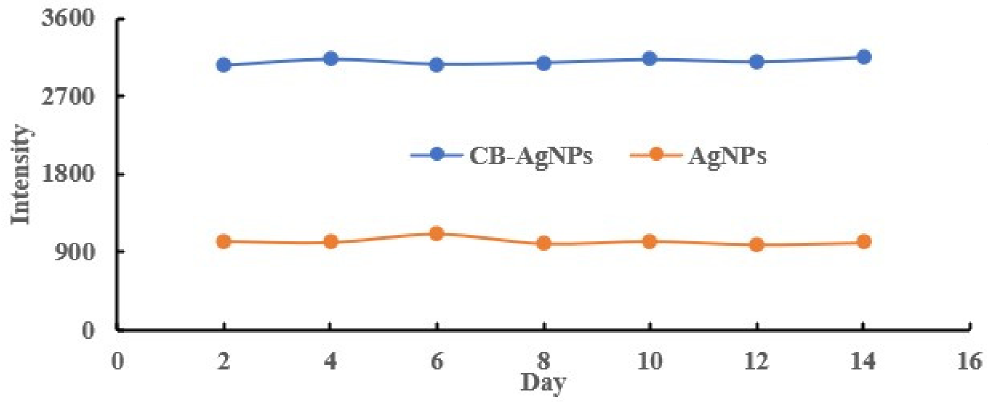

A

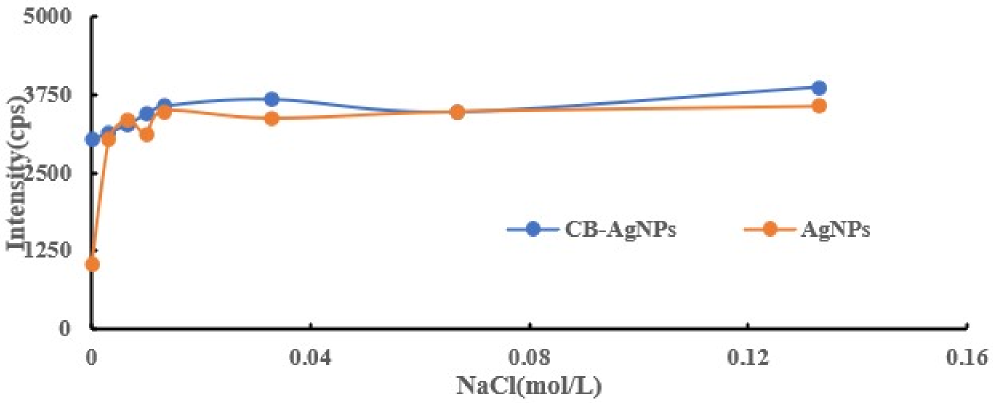

B

Figure S1. Stability of AgNPs and CB@AgNPs. **A**: The RRS signal of AgNPs and CB@AgNPs varying with time; **B**: The RRS signal of AgNPs and CB@AgNPs varying with NaCl.

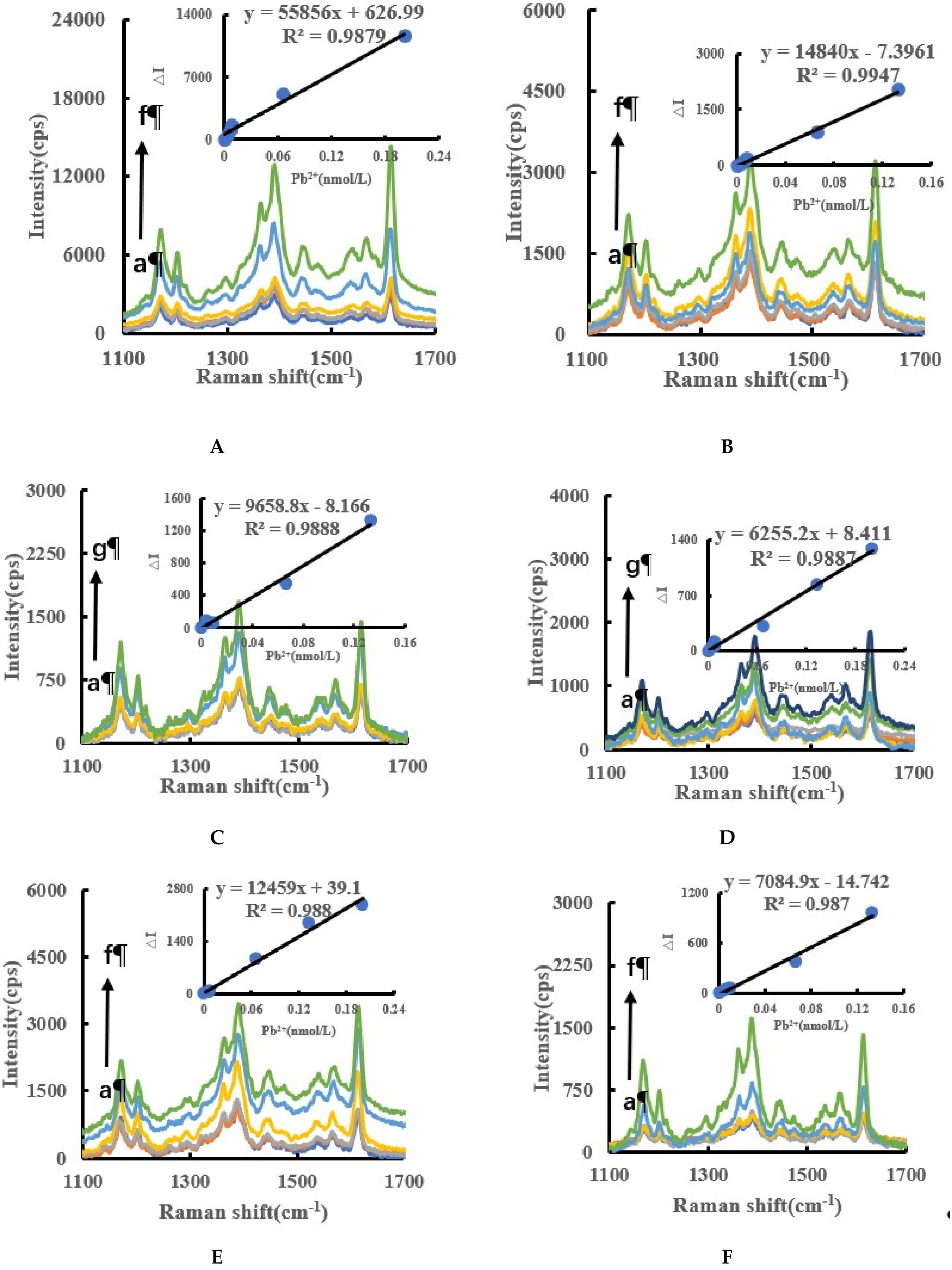

Figure S2. SERS spectra of AgNPs /LC -Fo-AgNO$_3$-Apt-inorganic pollutants system. **A**: a-f: (0, 4.47×10^{-3}, 6.7×10^{-3}, 8.94×10^{-3}, 6.7×10^{-2}, 0.133) nmol/L Pb^{2+} +0.667 nmol/L Apt$_{Pb}$+73.98 μmol/L NaAc-

HAc+1.33 μmol/L AgNPs +1.33 mmol/L $AgNO_3$+0.1 mol/L Fo+0.67 μmol/L VB4r+0.067 mol/L NaCl; **B**: a-f: (0, 4.47×10^{-3}, 6.7×10^{-3}, 8.94×10^{-3}, 6.7×10^{-2}, 0.133) nmol/L Pb^{2+} +0.667 nmol/L Apt_{Pb} +73.98 μmol/L NaAc-HAc+1.33 μmol/L CB +1.33 mmol/L $AgNO_3$+0.1 mol/L Fo+0.67 μmol/L VB4r+0.067 mol/L NaCl; **C**: a-f: (0, 4.47×10^{-3}, 6.7×10^{-3}, 8.94×10^{-3}, 6.7×10^{-2}, 0.133) nmol/L Pb^{2+} +0.667 nmol/L Apt_{Pb} +73.98 μmol/L NaAc-HAc+1.33 μmol/L OA+1.33 mmol/L $AgNO_3$+0.1 mol/L Fo+0.67 μmol/L VB4r+0.067 mol/L NaCl; **D**: a-g: (0, 4.47×10^{-3}, 6.7×10^{-3}, 8.94×10^{-3}, 6.7×10^{-2}, 0.133, 0.201) nmol/L Pb^{2+} +0.667 nmol/L Apt_{Pb} +73.98 μmol/L NaAc-HAc+1.33 μmol/L CB@AgNPs+1.33 mmol/L $AgNO_3$+0.1 mol/L Fo+0.67 μmol/L VB4r+0.067 mol/L NaCl; **E**: a-f: (0, 4.47×10^{-3}, 6.7×10^{-3}, 6.7×10^{-2}, 0.133, 0.201) nmol/L Pb^{2+} +0.667 nmol/L Apt_{Pb} +73.98 μmol/L NaAc-HAc+1.33 μmol/L DB+1.33 mmol/L $AgNO_3$+0.1 mol/L Fo+0.67 μmol/L VB4r+0.067 mol/L NaCl. ; **F**: a-f: (00, 4.47×10^{-3}, 6.7×10^{-3}, 8.94×10^{-3}, 6.7×10^{-2}, 0.133) nmol/L Pb^{2+} +0.667 nmol/L Apt_{Pb} +73.98 μmol/L NaAc-HAc+1.33 μmol/L DE+1.33 mmol/L $AgNO_3$+0.1 mol/L Fo+0.67 μmol/L VB4r+0.067 mol/L NaCl.

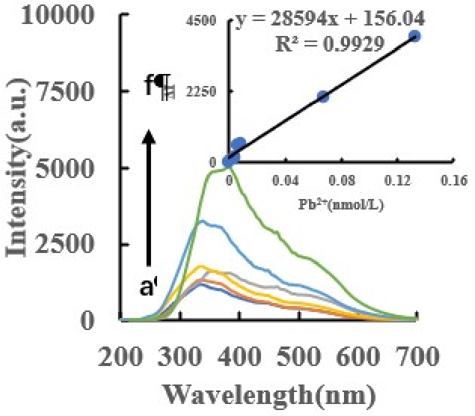

A

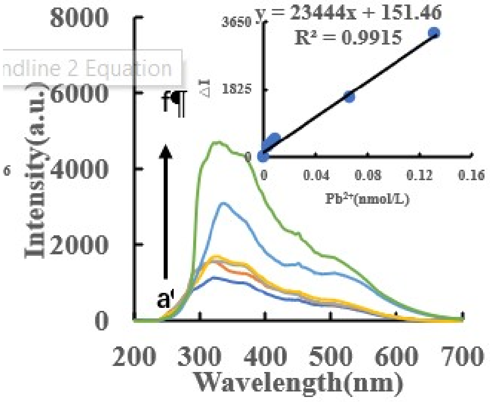

B

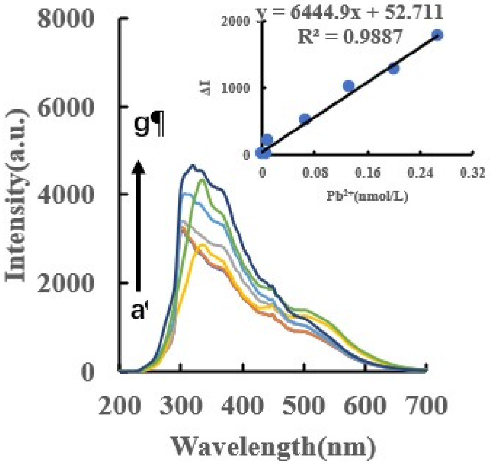

C

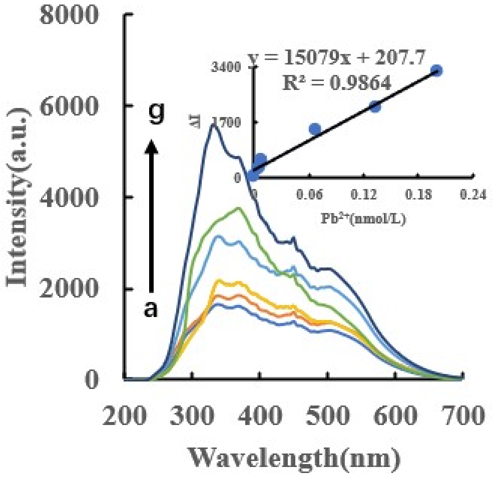

D

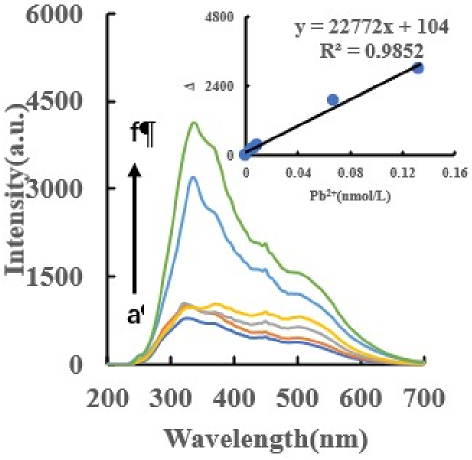

E

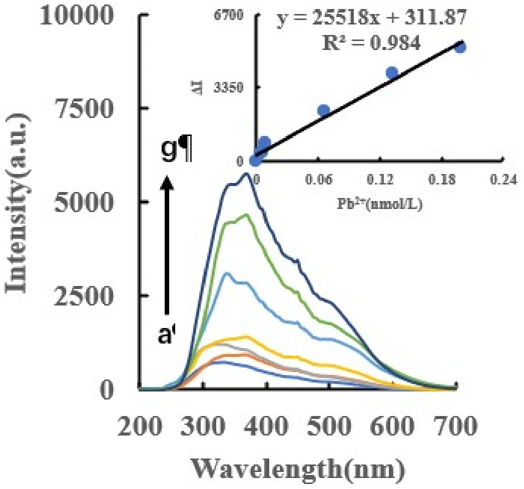

F

Figure S3. RRS spectra of AgNPs/LC-Fo-$AgNO_3$-Apt- inorganic pollutants system. **A**: a-f: (0, 4.47×10^{-3}, 6.7×10^{-3}, 8.94×10^{-3}, 6.7×10^{-2}, 0.133) nmol/L Pb^{2+} +0.667 nmol/L Apt_{Pb}+73.98 μmol/L NaAc-HAc+1.33 μmol/L AgNPs+1 mmol/L $AgNO_3$+0.1 mol/L Fo; **B**: a-f: (0, 4.47×10^{-3}, 6.7×10^{-3}, 8.94×10^{-3}, 6.7×10^{-2}, 0.133) nmol/L Pb^{2+} +0.667 nmol/L Apt_{Pb}+73.98 μmol/L NaAc-HAc+1.33 μmol/L CB +1 mmol/L $AgNO_3$+0.1 mol/L Fo; **C**: a-g: (0, 6.7×10^{-3}, 8.94×10^{-3}, 6.7×10^{-2}, 0.133, 0.201, 0.267) nmol/L Pb^{2+} +0.667 nmol/L Apt_{Pb} ++0.667 nmol/L Apt_{Pb}+73.98 μmol/L NaAc-HAc+1.33 μmol/L OA+1 mmol/L

$AgNO_3$+0.1 mol/L Fo; **D**: a-g: (0, 4.47×10^{-3}, 6.7×10^{-3}, 8.94×10^{-3}, 6.7×10^{-2}, 0.133, 0.201) nmol/L Pb^{2+} +0.667 nmol/L Apt_{Pb} + +73.98 μmol/L NaAc-HAc+1.33 μmol/L HA+1 mmol/L $AgNO_3$+0.1 mol/L Fo;. **E**: a-f: (0, 4.47×10^{-3}, 6.7×10^{-3}, 8.94×10^{-3}, 6.7×10^{-2}, 0.133) nmol/L Pb^{2+} +0.667 nmol/L Apt_{Pb} + +73.98 μmol/L NaAc-HAc+1.33 μmol/L DB+1 mmol/L $AgNO_3$+0.1 mol/L Fo;.**F**: a-g: (0, 4.47×10^{-3}, 6.7×10^{-3}, 8.94×10^{-3}, 6.7×10^{-2}, 0.133, 0.201) nmol/L Pb^{2+} +0.667 nmol/L Apt_{Pb} + +73.98 μmol/L NaAc-HAc+1.33 μmol/L DE+1 mmol/L $AgNO_3$+0.1 mol/L Fo.

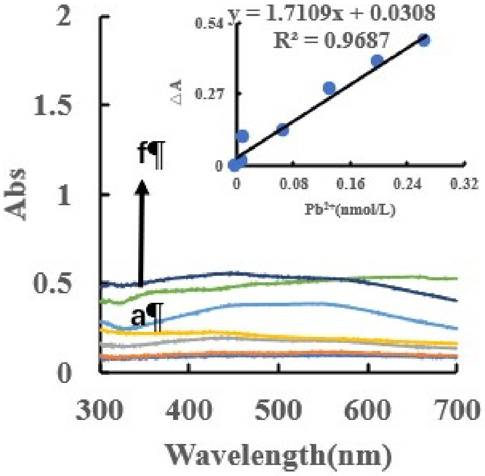

A

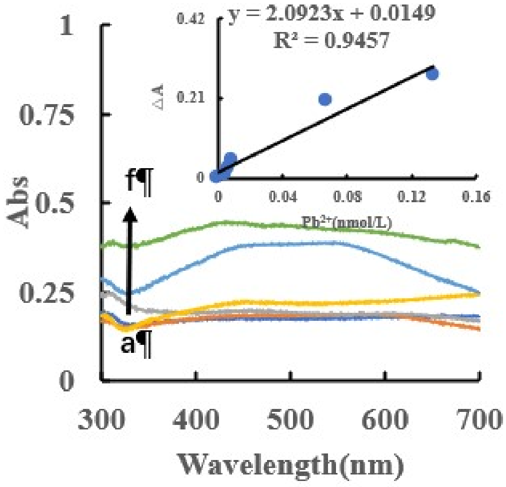

B

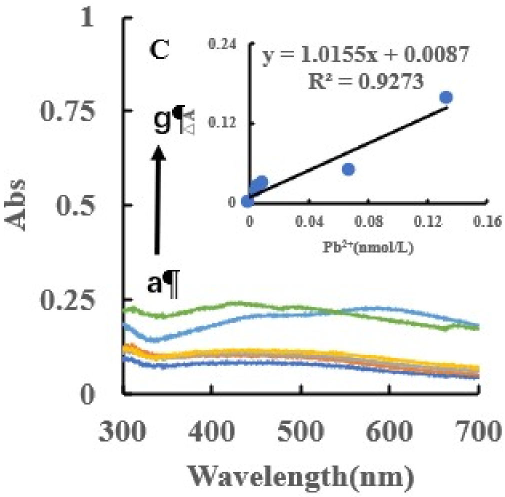

C

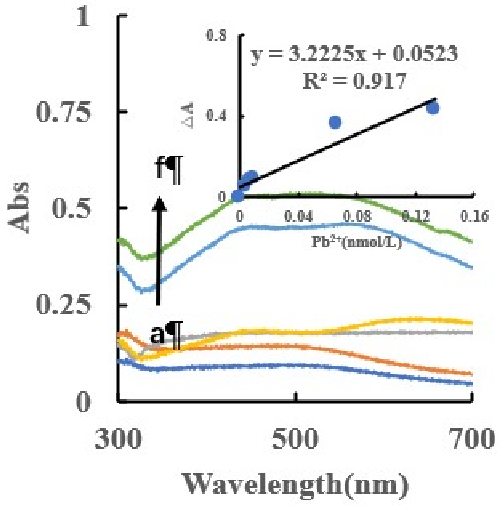

D

Figure S4. Abs spectra of Apt_{Pb} AgNPs/LC-Fo-$AgNO_3$-Pb^{2+} system. **A**: a-f: (0, 6.7×10^{-3}, 8.94×10^{-3}, 6.7×10^{-2}, 0.133, 0.201, 0.267) nmol/L Pb^{2+} +0.667 nmol/L Apt_{Pb}+73.98 μmol/L NaAc-HAc+1.33 μmol/L OA+1 mmol/L $AgNO_3$+0.1 mol/L Fo; **B**: a-f: (0, 4.47×10^{-3}, 6.7×10^{-3}, 8.94×10^{-3}, 6.7×10^{-2}, 0.133) nmol/L Pb^{2+} +0.667 nmol/L Apt_{Pb}+73.98 μmol/L NaAc-HAc+1.33 μmol/L HA +1 mmol/L $AgNO_3$+0.1 mol/L Fo; **C**: a-g: (0, 4.47×10^{-3}, 6.7×10^{-3}, 8.94×10^{-3}, 6.7×10^{-2}, 0.133) nmol/L Pb^{2+} +0.667 nmol/L Apt_{Pb} ++0.667 nmol/L Apt_{Pb}+73.98 μmol/L NaAc-HAc+1.33 μmol/L DB+1 mmol/L $AgNO_3$+0.1 mol/L Fo; **D**: a-g: (0, 4.47×10^{-3}, 6.7×10^{-3}, 8.94×10^{-3}, 6.7×10^{-2}, 0.133) nmol/L Pb^{2+} +0.667 nmol/L Apt_{Pb} + +73.98 μmol/L NaAc-HAc+1.33 μmol/L DE+1 mmol/L $AgNO_3$+0.1 mol/L Fo.

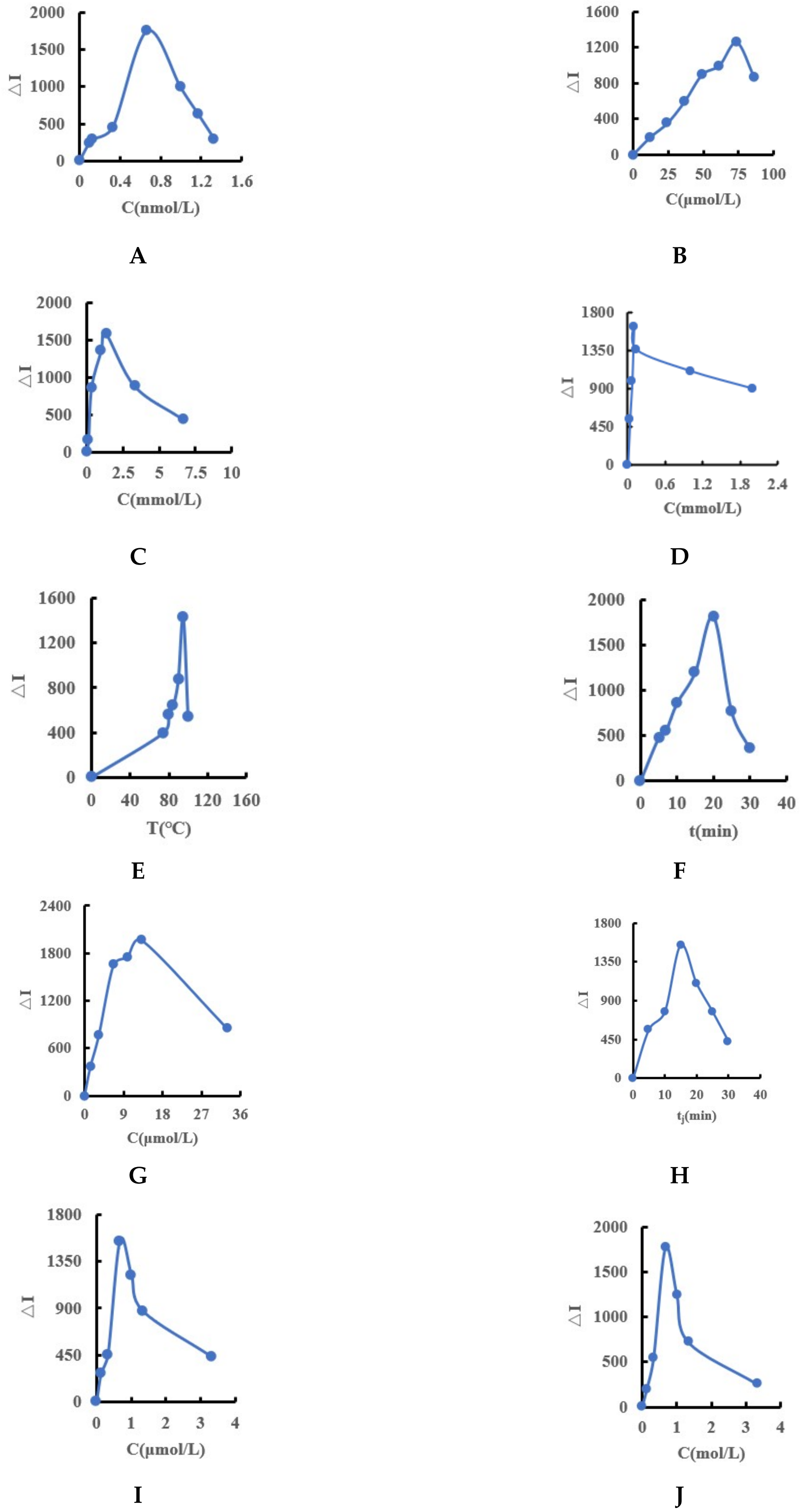

Figure S5. Optimization of analysis conditions. **A**: Apt_{Pb}; **B**: NaAc-HAc; **C**: $AgNO_3$; **D**: Fo; **E**: reaction temperature; **F**: reaction time; **G**: CB@AgNPs; **H**: standing time; **I**: NaCl; **J**: VB4r.

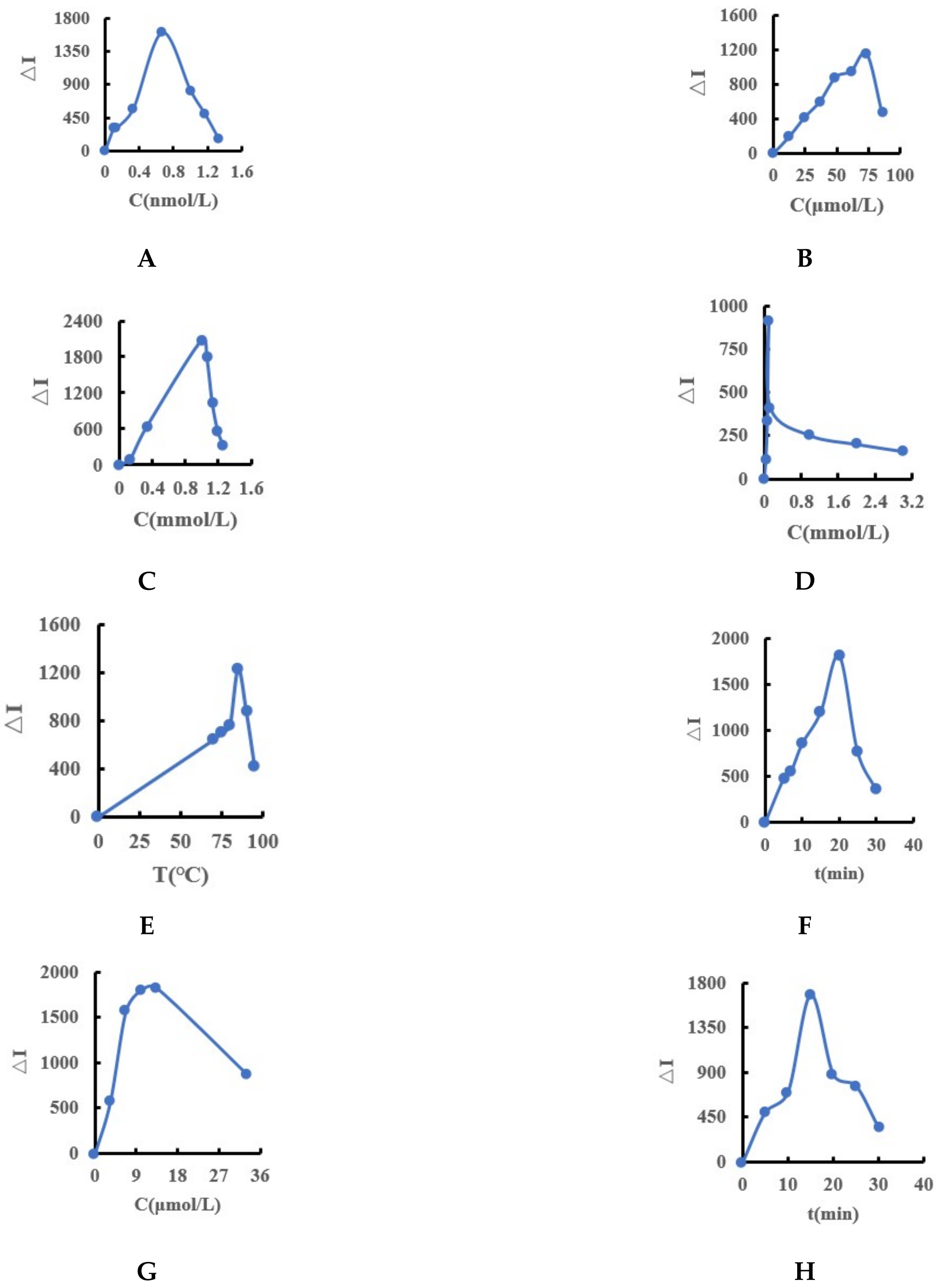

Figure S6. Optimization of RRS analysis conditions. **A**: Apt_{Pb}; **B**: NaAc-HAc; **C**: $AgNO_3$; **D**: Fo; **E**: reaction temperature; **F**: reaction time; **G**: CB@AgNPs; **H**: standing time;.

Table S1. Comparison of LC/CB@AgNPs/AgNPs catalytic action and inhibitory action of aptamers.

LC types	Working curve	Linear range	Coefficient(R^2)
CB	$\Delta I = 1382.2C_{CB}-62.7$	0.01–0.1mmol/L	0.9983
Apt_{Pb} -CB	$\Delta I = 1000.3C_{Apt}-13.4$	0.0667–0.667 nmol/L	0.9591
OA	$\Delta I = 2895.6C_{OA}-560.1$	0.001–0.01 mmol/L	0.91579
Apt_{Pb} -OA	$\Delta I = 1282.7C_{Apt}-486.2$	0.0667–0.667 nmol/L	0.90515
HA	$\Delta I = 2245.5C_{HA}-251.8$	0.001–0.01 mmol/L	0.9383
Apt_{Pb} -HA	$\Delta I = 3844.5C_{Apt}-25.1$	0.0667–0.667 nmol/L	0.9334
DB	$\Delta I=1072.6C_{DB}- 219.5$	0.001–0.01 mmol/L	0.90667
Apt_{Pb} -DB	$\Delta I=1640.7C_{Apt}- 1193.9$	0.0667–0.667 nmol/L	0.9132
DE	$\Delta I= 748.4C_{DE}- 223.2$	0.001–0.01 mmol/L	0.9223
Apt_{Pb} -DE	$\Delta I= 1142.7C_{Apt}- 594.6$	0.0667–0.667 nmol/L	0.9168
CB@AgNPs	$\Delta I = 9776.2C_{CB@AgNPs} +5.4$	0.01–0.133 mmol/L	0.9991
AptPb - CB@AgNPs	$\Delta I= 7983C_{Apt}- 278.4$	0.0667–0.667 nmol/L	0.9868
AgNPs	$\Delta I = 5397.5C_{AgNPs} +1.7$	0.01–0.133 mmol/L	0.9963
Apt_{Pb} -AgNPs	$\Delta I= 4378C_{Apt}- 304.6$	0.0667–0.667 nmol/L	0.9831

Table S2. Analysis characteristics of Pb^{2+} detection by SERS/RRS/Abs.

LCs	Method	Linear range (nmol/L)	Working curve	Coefficient	LD (nmol/L)
CB@AgNPs	SERS	4.47×10^{-3}–0.201	$\Delta I_{1618cm-1}= 76201C- 414.6$	0.998	3×10^{-3}
	RRS	4.47×10^{-3}–0.201	$\Delta I_{370nm}= 33687C+ 166$	0.996	3×10^{-3}
	Abs	4.47×10^{-2}–0.133	$\Delta A_{430nm}= 4.7887C+0.001$	0.9947	2×10^{-2}
AgNPs	SERS	4.47×10^{-3}–0.133	$\Delta I_{1618cm-1}= 55856C+627$	0.9879	3×10^{-3}
	RRS	4.47×10^{-3}–0.133	$\Delta I_{370nm}= 28594C+ 156$	0.9929	3×10^{-3}
	Abs	4.47×10^{-2}–0.133	$\Delta A_{430nm}= 3.9979C+0.05$	0.986	2×10^{-2}
CB	SERS	4.47×10^{-3}–0.133	$\Delta I_{1618cm-1}= 14840C- 13.2$	0.9947	3×10^{-3}
	RRS	8.94×10^{-3}–0.133	$\Delta I_{370nm}= 23444C+ 151.5$	0.9915	5×10^{-3}
	Abs	4.47×10^{-2}–0.133	$\Delta A_{430nm}= 3.9143C+0.03$	0.9808	2×10^{-2}
OA	SERS	8.94×10^{-3}–0.133	$\Delta I_{1618cm-1}= 9658.8C-8.2$	0.9888	5×10^{-3}
	RRS	8.94×10^{-3}–0.267	$\Delta I_{370nm}=6444.9C+ 52.7$	0.9887	5×10^{-3}
	Abs	6.7×10^{-2}–0.267	$\Delta A_{450nm}= 1.7109C+ 0.03$	0.9687	3×10^{-2}
HA	SERS	8.94×10^{-3}–0.201	$\Delta I_{1618cm-1}= 6255.2C+ 8.4$	0.9887	5×10^{-3}
	RRS	8.94×10^{-3}–0.201	$\Delta I_{370nm}=15079C+ 207.7$	0.9864	5×10^{-3}
	Abs	4.47×10^{-2}–0.133	$\Delta A_{450nm}= 2.10C+ 0.02$	0.9457	2×10^{-2}
DB	SERS	8.94×10^{-3}–0.201	$\Delta I_{1618cm-1}= 12459C+39.1$	0.988	4×10^{-3}
	RRS	8.94×10^{-3}–0.133	$\Delta I_{370nm}=22772C+104$	0.9852	5×10^{-3}
	Abs	4.47×10^{-2}–0.133	$\Delta A_{440nm}=1.0C+ 0.009$	0.9273	2×10^{-2}
DE	SERS	8.94×10^{-3}–0.133	$\Delta I_{1618cm-1}= 7084.9C-14.7$	0.987	5×10^{-3}
	RRS	8.94×10^{-3}–0.201	$\Delta I_{370nm}=25518C+311.9$	0.984	6×10^{-3}
	Abs	6.7×10^{-2}–0.133	$\Delta A_{440nm}=3.20C+ 0.05$	0.917	3×10^{-2}

Table S3. The analysis characteristics of inorganic pollutants by Apt-CB@AgNPs catalytic SERS/RRS.

Analytes	Method	Linear range (nmol/L)	Working curve	Coefficient	LD (nmol/L)
Pb^{2+}	SERS	4.47×10^{-3}–0.201	$\Delta I_{1618cm-1}$= 76201C− 414.6	0.998	3×10^{-3}
	RRS	4.47×10^{-3}–0.201	ΔI_{370nm}= 33687C+ 166	0.996	3×10^{-3}
As^{3+}	SERS	6.67×10^{-3}–0.133	$\Delta I_{1618cm-1}$= 51005C-92.4	0.9877	3×10^{-3}
	RRS	6.67×10^{-3}–0.133	ΔI_{370nm}=29280C+ 40.2	0.9884	3×10^{-3}
Cd^{2+}	SERS	6.67×10^{-3}–0.133	$\Delta I_{1618cm-1}$= 60283C-187.5	0.9811	3×10^{-3}
	RRS	6.67×10^{-3}–0.133	ΔI_{370nm}=32880C+ 132.7	0.9874	3×10^{-3}
Hg^{2+}	SERS	0.67–30	$\Delta I_{1618cm-1}$= 228.28C+ 197.8	0.9807	3
	RRS	0.67–30	ΔI_{370nm}=211.19C+ 343.3	0.9867	3

Table S4. Comparison of analysis characteristics between this method and the reported method.

Method*	Method principle	Linear range /（nmol/L）	DL/（nmol/L）	Annotation	Ref.
FL	The functional groups on the surface of fluorescent nanoparticles combined with Pb^{2+} to enhance FL.	5–50	3	The stability is good but the detection range is not wide.	[37]
PE	Pb^{2+} bind to the captured DNA in the detection electrode to cause signal changes.	0.5–900	0.166	High sensitivity but cumbersome substrate synthesis.	[38]
FL	The combination of Pb^{2+} with the AuNP-DNA probe in the detection electrode caused a signal change.	0–50	2.5	Good selectivity but complicated electrode modification.	[39]
SERS	When Pb^{2+} bind to ARS, colloidal clusters with high SERS activity were generated, resulting in enhanced Raman signal.	8–20000	6	Fast and convenient but low detection sensitivity.	[40]
SERS	Pb(II) and Apt specifically bint to form a G-tetrad structure, and the combination of Pb(II)-Apt tetrad structure and CDAu produced a strong Raman effect.	1.7–13.3	0.8	The selectivity is good, but the operation is more complicated.	[41]
Abs	TpPapd-Apt-Pb^{2+} was combined with the DNA reaction of heme (HM), and there was a surface plasmon resonance (SPR) absorption peak at 395nm. An analytical method for the determination of lead ions had been established.	0.001–0.1	0.004	High sensitivity, fast speed and good selectivity, but the operation is more complicated.	[25]
ECL	Pb^{2+} was captured by Apt 1-PtNPs and formed a G-quadruplex, and then made PtNPs close enough to	0.1–1000	0.037	The detection range is high, but the stability is poor.	[42]

	CdTe QD to cause ECL intensity changes.				
SERS	The Apt combined with Pb^{2+} to form a complex, detached from the surface of CB@AgNPs, and restored its catalysis, which enhanced the SERS signal	0.0047–0.201	0.0036	Sensitive, simple and fast.	This assay

* FL-fluorescence, PE- photoelectrochemical, ECL-electrochemiluminescence.

Table S5. The influence of interfering ions on the system.

Interfering ion	Relative multiple	Relative error (%)	Interfering ion	Relative multiple	Relative error (%)
Mg^{2+}	1000	2.4	Ca^{2+}	1000	2.9
Fe^{3+}	100	−1.9	Zn^{2+}	500	−2.8
Cu^{2+}	100	−6.3	Al^{3+}	500	−7.1
Co^{2+}	500	−1.4	Hg^{2+}	100	3.0
Ba^{2+}	1000	−5.9	NH^{4+}	500	1.3
Fe^{2+}	1000	3.4	Mn^{2+}	1000	4.5
Cr^{6+}	1000	2.1	Cr^{3+}	1000	−3.1
NO_2^-	100	−1.4	PO_4^{3-}	100	−5.5
serum protein $^-$	500	−2.7	HSA	100	−1.5
BSA^-	1000	3.6	ascorbic acid	500	1.9

Table S6. SERS measurement results of the samples.

Sample	Average （nmol/L）	Added Pb^{2+} （nmol/L）	Found （nmol/L）	Recovery (%)	RSD （%）	Content (nmol/L or ng/g)
Water 1	0.1934	0.067	0.2565	94.18	5.4	0.1945
Water 2	0.1801	0.067	0.2534	109.4	7.1	0.1815
Water 3	0.1843	0.067	0.2556	107.8	4.3	0.1857
Water 4	0.3295	0.067	0.3989	103.6	6.1	0.3310
Preserved eggs 1	0.4287	0.067	0.4995	105.67	1.3	14.796
Preserved eggs 2	0.4659	0.067	0.5298	95.37	2.9	15.173
Preserved eggs 3	0.4778	0.067	0.5482	105.07	3.5	15.389
Preserved eggs 4	0.5003	0.067	0.5623	92.53	2.5	16.653
Orange peel 1	0.1003	0.067	0.1632	93.88	3.2	4.986
Orange peel 2	0.1196	0.067	0.1856	98.51	6.4	5.352
Orange peel 3	0.1284	0.067	0.1987	104.9	5.1	5.435
Orange peel 4	0.1467	0.067	0.2156	102.8	2.9	5.683
